# Development of a Hydrazine-Based Solid-Phase Extraction and Clean-Up Method for Highly Selective Quantification of Zearalenone in Edible Vegetable Oils by HPLC-FLD

**DOI:** 10.3390/toxins14080549

**Published:** 2022-08-11

**Authors:** Matthias Koch, Tatjana Mauch, Juliane Riedel

**Affiliations:** Division of Organic Trace and Food Analysis, Department of Analytical Chemistry, Reference Materials, Bundesanstalt für Materialforschung und -prüfung (BAM), Richard-Willstätter-Str. 11, D-12489 Berlin, Germany

**Keywords:** mycotoxin, food, reversible covalent hydrazine chemistry (RCHC), quantitative determination

## Abstract

Rapid, cost-efficient, and eco-friendly methods are desired today for routine analysis of the *Fusarium* mycotoxin zearalenone (ZEN) in edible vegetable oils. Liquid chromatography with fluorescence detection (HPLC-FLD) is commonly used to reliably control the specified ZEN maximum levels, which requires efficient sample clean-up to avoid matrix interferences. Therefore, a highly selective extraction and clean-up method based on reversible covalent hydrazine chemistry (RCHC) using hydrazine-functionalized silica was developed. This efficient solid-phase extraction (SPE) involves reversible hydrazone formation of ZEN with the hydrazine moiety covalently bound to a solid phase. Optimal conditions were achieved with 1 mL SPE cartridges filled with 400 mg of hydrazine-functionalized silica. The developed RCHC-SPE method was validated in an interlaboratory comparison study (ILC) with twelve participants analyzing six edible vegetable oils with a focus on maize oils. The derived method parameters (ZEN recovery 83%, repeatability 7.0%, and reproducibility 18%) meet the performance criteria of Commission Regulation (EC) No 401/2006. The developed RCHC-SPE-based HPLC-FLD method allows the reliable quantification of ZEN in the range of 47–494 µg/kg for different types of edible vegetable oils, also for matrix-reach native oils. Due to the high efficiency, the significantly reduced matrix load helps to extend the lifetime of analytical equipment. Furthermore, the re-useability of the RCHC-SPE cartridges contributes to an eco-friendly approach and reduced analysis costs. To our knowledge, this is the first report on ZEN quantification in edible vegetable oils based on manual RCHC-SPE cartridges. Due to its high performance, the developed RCHC-SPE method is a promising alternative to the current European standard method EN 16924:2017 (HPLC-FLD part).

## 1. Introduction

Zearalenone (ZEN) is one of the top five agriculturally important mycotoxins contaminating food and feed worldwide [1]. ZEN is produced by several *Fusarium* species including *F. graminearum* (*Gibberella zeae*), *F. culmorum*, *F. cerealis*, and *F. equiseti* [2]. In vitro and in vivo studies demonstrate ZEN to be estrogenic, hepatotoxic, immunotoxic, and carcinogenic [3]. A variety of relevant crops and products thereof can be contaminated by ZEN including barley, oats, wheat, sorghum, rice, and maize. Due to its lipophilic property, ZEN can be enriched in vegetable oils, contributing considerably to the overall ZEN exposure [4]. Maize germ oils, in particular, could be a significant source of ZEN, as evidence from several decades suggests [4,5,6,7,8]. As a consequence, a maximum level of 400 µg/kg ZEN for refined maize oil was established in the European Union [9].

Several methods have been reported for the quantification of ZEN in edible oils, mostly based on high-performance liquid chromatography (HPLC) [4]. HPLC coupled to tandem mass spectrometry (HPLC-MS/MS) has gained increasing importance not only for ZEN but also for sensitive and selective analysis of other mycotoxins and organic contaminants. However, HPLC with fluorescence detection (FLD) is still commonly used for routine analysis because of its broad availability combined with low acquisition costs and maintenance costs. Both detection techniques (MS, FLD) are implemented in the European standard procedure EN 16924:2017 for analysis of ZEN in edible vegetable oils [10], which can be used alternatively. The natural fluorescence of ZEN principally allows the use of HPLC-FLD but requires an efficient clean-up, especially for native oils, to ensure a reliable ZEN quantification without interfering matrix compounds. Sample preparation methods such as gel permeation chromatography (GPC) or immunoaffinity column (IAC) clean-up [11] and liquid–liquid extraction (LLE) [12] have been reported. However, these techniques lack from laborious handling and costs (GPC, IAC) or have poor selectivity (LLE). Consequently, LLE with subsequent HPLC-FLD analysis as applied in EN 16924:2017 is susceptible for matrix-rich samples (native oils) posing a risk for non-quantifiable results.

**Figure 1:**
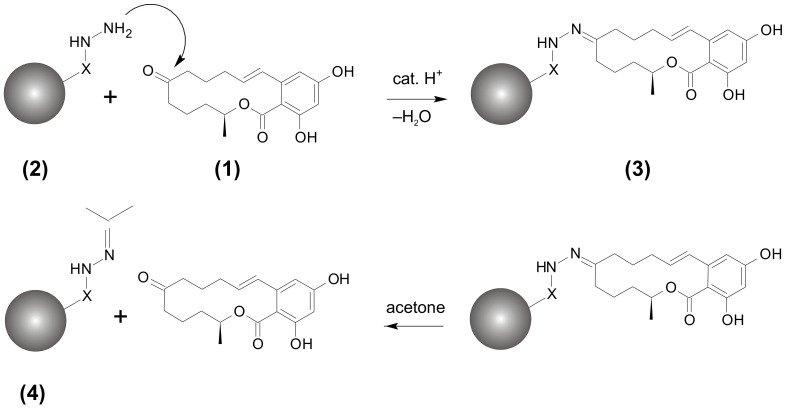
Concept of reversible covalent hydrazine chemistry (RCHC): Zearalenone (ZEN) (**1**) is extracted from oil sample by reaction with the hydrazine moiety linked to a solid phase (**2**) to form ZEN-hydrazone (**3**), which can be cleaved into ZEN after removal of matrix compounds. The acetone-loaded solid phase (**4**) can be re-activated to (**2**) for its reuse.

To overcome these shortcomings, in particular the poor selectivity of LLE, the concept of reversible covalent hydrazine chemistry (RCHC) is favored. The RCHC approach was shown to be an efficient technique for extraction and clean-up of ZEN from edible oils [13,14,15]. RCHC represents a solid-phase extraction (SPE) retaining the analyte by formation of a covalent bond rather than unspecific physisorption. ZEN contains an isolated keto group that readily reacts with the hydrazine groups immobilized on a solid phase to form a ZEN-hydrazone (Figure 1). Once ZEN is covalently bound to the solid phase, the oil matrix can be removed by several washing steps. After clean-up, ZEN is released by a cleaving step and analyzed by HPLC-FLD.

While we developed an online RCHC-SPE coupling to HPLC-FLD for the automated analysis of ZEN in edible oils using pressure-stable hydrazine-functionalized silica [14], our first manual RCHC application was based on a batch-design using bulk particles of hydrazine-functionalized polymers [13]. Although very efficient, the different sample preparation steps (coupling, clean-up with several adding and removal steps of solvents, drying, cleavage, transfer) require careful handling to avoid losses of the hydrazine-functionalized material, making the manual RCHC approach unfavorable for routine analyses.

Thus, the aim of the present study was to develop a selective, reliable, and also cost-efficient method for routine analysis of ZEN in edible oils based on the RCHC technique and HPLC-FLD. The focus was placed on preparation and optimization of a ready-to-use RCHC-SPE cartridge for a time- and cost-saving sample preparation. Method validation was performed with respect to the performance criteria of Regulation (EC) No 401/2006 [16] regarding ZEN recovery, repeatability, and reproducibility by conducting an interlaboratory comparison study (ILC) with 12 expert laboratories. While covalent hydrazine chemistry for ZEN analysis has been reported previously, this is the first study to focus on manual RCHC-SPE cartridges, which are user-friendly and eco-friendly to handle.

## 2. Results and Discussion

### 2.1. Selection of a Hydrazine-Functionalized Material

Nine hydrazine-functionalized materials (HFM) were tested for their suitability as SPE-phase consisting of silica or a polymer backbone (Table 1). Two of these HFM were also included in previous studies: SC-1, a tosyl hydrazine silica material, was used to develop an SPE-HPLC online-coupling method [14]. PS-1, a polymer-bound sulfonyl hydrazine consisting of macroporous polystyrene-divinylbenzene (PS/DVB), was applied for the first RCHC batch method [13].

The initial evaluation of the nine HFM was focused on their property for coupling of ZEN to ensure acceptable processing times that are as short as possible while maintaining high ZEN recovery. It was found that an increase in ZEN coupling rate was possible compared to PS-1 as the first material used (Table 1). Both the synthesized materials PS-4 and PS-5, but especially the silica-based hydrazine material SC-1, showed higher coupling rates. The five HFM with the highest coupling constants (SC-1, SC-3, PS-1, PS-4, and PS-5) were subsequently used to assess the properties in more detail. The results in Table 2 for coupling, decoupling, and ZEN recovery of the five HFM candidates were derived by using default conditions based on [13]: For coupling, 100 mg of HFM was weighed into a 2 mL Eppendorf safelock tube. After adding 1 mL of ZEN solution (100 ng/mL) in methanol (MeOH), the safelock tube was shaken on a horizontal shaker at 250 min^−1^. Subsequently, the tube was centrifuged at 14,500 rpm (~14,000× *g*). The supernatant was taken off and analyzed by HPLC-FLD. For decoupling, 100 mg of ZEN-loaded HFM was placed into a 2 mL safelock tube. After adding 1 mL of cleaving solution acetone:0.13 M hydrochloric acid (HCl) 70/30 *v*/*v*, the safelock tube was shaken on a horizontal shaker. Subsequently, the tube was centrifuged and the supernatant was removed for HPLC-FLD analysis.

It was shown that the applied amount of ZEN (100 ng) was coupled to each of the five HFM after 2 h, with SC-1 giving the best result and confirming the outcome of the initial study. In addition, SC-1 also revealed the fastest cleaving behavior. High ZEN recoveries of 95% after cleavage were achieved for SC-1 and SC-3, with final recovery for SC-1 already completed after 30 min. Other advantages of SC-1 are that it does not tend to swell (important for SPE cartridge format), is not compressible, and has consistent quality across all batches tested. In summary, SC-1 was selected for RCHC-SPE development due to its superior coupling, decoupling, and ZEN recovery properties.

### 2.2. Preparation of an RCHC-SPE Cartridge

**Figure 2:**
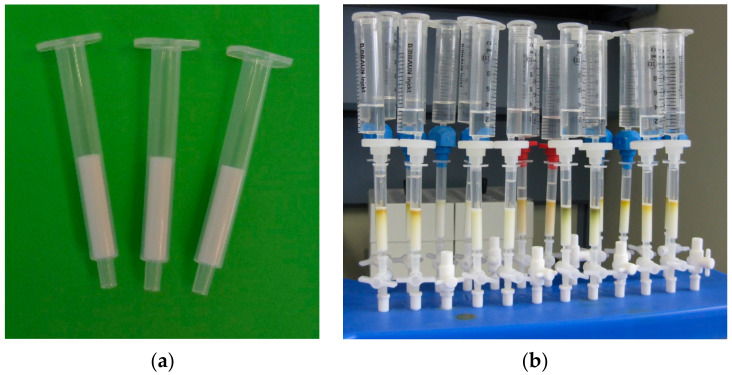
(**a**) RCHC-SPE cartridges: 1 mL PP cartridges filled with 400 ± 2 mg hydrazine-functionalized material SC-1; (**b**) RCHC-SPE cartridges in use for extraction and clean-up of ZEN from edible vegetable oils.

User-friendly handling of a ready-to-use cartridge was one of the main objectives of the concept. Therefore, commercial cartridge format (empty polypropylene (PP) cartridges with 1, 3, and 6 mL, Macherey & Nagel, Düren, Germany) were used that fit easily on conventional SPE vacuum manifolds. Since each cartridge was calculated to contain a minimum of 100 mg and a maximum of 500 mg, only the small version (1 mL cartridge) ensured a significant fill level. The final assignment of 400 ± 2 mg of SC-1 per cartridge was based on the results of an optimization process considering ZEN recovery and processing time. The HFM was placed between an upper and lower polyethylene (PE) frit. The RCHC-SPE cartridges (Figure 2) filled with SC-1 are stable for at least 12 months at 4 °C storage.

### 2.3. RCHC-SPE Method Development

**Figure 3:**
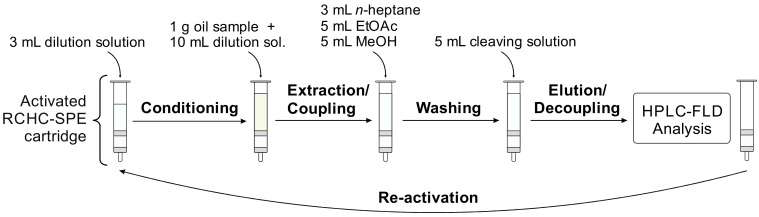
Scheme for extraction and clean-up of ZEN from edible vegetable oils using RCHC-SPE cartridge.

Based on the initial results, the most-suited HFM (SC-1) was selected for RCHC-SPE development and optimization of the individual process steps: activation (and re-activation) of the SC-1 material, ZEN extraction from edible vegetable oils (coupling), removal of oil matrix (clean-up), and ZEN release from RCHC-SPE cartridge (cleavage/decoupling). The optimized procedure is displayed in Figure 3.

*Activation and re-activation*: Our previous studies [13,14] already showed that special attention must be paid to the activation of the unused HFM before use, as this increases the efficiency of the coupling process. Appropriate activation of the hydrazine group is achieved with a solution containing HCl to form the protonated species R-NH-NH_3_^+^ Cl^−^. The optimized activation of SC-1 was as follows: 40 g of new HFM (sufficient for 100 RCHC-SPE cartridges) wase put into a glass column and eluted with 800 mL of methanol:0.4M HCl 90:10 *v*/*v* overnight. Subsequently, 150 mL of methanol and 100 mL of diethyl ether were passed through the column. The HFM was dried in the gentle nitrogen stream and stored at 4 °C. RCHC-SPE cartridges can be re-activated after use by applying the same procedure as performed for initial activation of bulk HFM. Re-activation reagents for one RCHC-SPE cartridge: 10 mL methanol:0.4 M HCl 90:10 *v*/*v*, 3 mL methanol, and 3 mL diethyl ether. During the re-activation process, acetone is released from the used RCHC-SPE and the activated hydrazine group is recovered (Figure 1).

*Conditioning*: The activated RCHC-SPE cartridge is connected to a common SPE vacuum manifold (Figure 2b). For conditioning of the solid phase, 3 mL of dilution solution (n-heptane:acetic acid, 99.9:0.1 *v*/*v*) was eluted with 2 mL/min. While acetic acid is to maintain protonation, n-heptane was chosen because it is needed for the next step (extraction).

*Extraction (coupling)*: To apply a viscous, non-polar oil to the RCHC-SPE cartridge, the sample has to be diluted by a non-polar, inert, and non-toxic solvent; n-heptane was tested as a suitable dilution solvent. The diluted edible vegetable oil sample (1 g oil mixed with 10 mL of dilution solution) was passed through the RCHC-SPE cartridge with a flow rate of 1 mL/min.

*Clean-up (washing)*: After coupling, the ZEN-loaded RCHC-SPE cartridge was consecutively washed with three different solvents of increasing polarity (flow rates 2 mL/min) for two reasons: since an aqueous solvent is required for the subsequent cleavage step, the solid phase must be transferred from a non-polar to a polar environment. In addition, a variety of matrix compounds can best be removed by using solvents of different polarities, starting with 3 mL of n-heptane, followed by 5 mL of ethyl acetate (EtOAc), and 5 mL of MeOH.

*Cleavage (decoupling)*: The cleaving is based on the displacement of ZEN from the hydrazine group by an excess of acetone. In our previous study [13], we already showed that a mixture of acetone and aqueous HCl is efficient for cleavage when PS-1 is used as HFM. However, the cleaving conditions using SC-1 had to be optimized regarding the acetone/HCl ratio (investigated range: 50:50 to 100:0 *v*/*v*) and the HCL concentration (tested: 0.1 M to 0.6 M). Best results were obtained using acetone:0.4 M HCL 95:5 *v*/*v*, which was selected as cleaving solution. After briefly drying the cartridge after the last washing step for 5 s in a gentle vacuum, ZEN was decoupled by a total of 5 mL of cleaving solution divided into two steps: after applying 2 mL with 1 mL/min, the flow was stopped for 30 min in order to complete the ZEN decoupling reaction. The remaining 3 mL of cleaving solution was passed through (1 mL/min), followed by drying the cartridge for 5 s in a light vacuum. The collected eluate was evaporated to dryness at 40 °C in a gentle stream of nitrogen, re-dissolved in 1 mL of acetonitrile:water:formic acid 50:50:0.1 (*v*/*v*/*v*), and transferred into an HPLC vial. The HPLC-FLD method used for measurement and quantification of ZEN is described in Section 4.4.

### 2.4. Interlaboratory Comparison Study

For the validation of the developed RCHC-SPE method, an interlaboratory comparison study (ILC) was conducted with 12 laboratories experienced in mycotoxin/ZEN analysis in food. To cover the range of the official standard method [10] including the EU maximum level, six edible vegetable oil samples were selected, with mass fractions between 50 and 500 µg/kg. In terms of prevailing occurrences and regulations, the focus was on maize oil samples.

Each participant received two bottles each of the six oil samples along with thirty-eight already prepared and activated RCHC-SPE cartridges and a detailed description of how to apply the RCHC-SPE method. To ensure proper calibration, each participant received a certified ZEN stock solution, which has to be used mandatorily. In order to control the calibration at participants site, the ILC organizer also prepared a control solution of ZEN in acetonitrile with a concentration unknown for the participants. With the exception of one participant, all other laboratories analyzed the ZEN control solution within ±15% of the target value of 200 ng/mL. The ILC results for the determination of ZEN in six edible vegetable oil samples are shown in Table 3.

The limit of quantification (LOQ) for ZEN in edible vegetable oils was reported by the ILC participants ranging from 1.5 µg/kg to 24 µg/kg. An LOQ value of 1.5 µg/kg was also determined in our in-house laboratory validation based on the German standard DIN 32645 using a calibration curve model. However, due to several ILC-participants stating LOQ values >5 µg/kg, sample-No. 6 was not considered for further data evaluation. Two of the twelve laboratories had to be excluded from further data processing due to technical problems that could not be solved in time. The mean values of the ILC samples displayed in Table 3 were calculated as the robust means (median) of the lab means taking into account six results from each participant (three replicates × two bottles). An individual ZEN recovery for each oil sample was derived by comparison of the ILC median with the ZEN value determined by the reference method SIDA-HPLC-MS/MS (SIDA: stable isotope dilution assay). The mean ZEN recovery of 83% obtained as the average of five recovery values displayed in Table 3 is in good agreement with the method performance requirements laid down in EC No 401/2006 [16], stating a ZEN recovery of 70–120% for ZEN contents >50 µg/kg. It is noteworthy that there is little variation in the recovery rate regardless of the ZEN content and the type of oil. The obtained repeatability (RSD_r_, mean 7.0%) and reproducibility (RSD_R_, mean 18%) also fulfill the performance criteria of EC No 401/2006 of ≤25% for RSD_r_ and ≤40% for RSD_R_. It should be emphasized that these performance parameters of the developed RCHC-SPE method are comparable to the official standard method EN 16924 (HPLC-FLD part).

**Figure 4:**
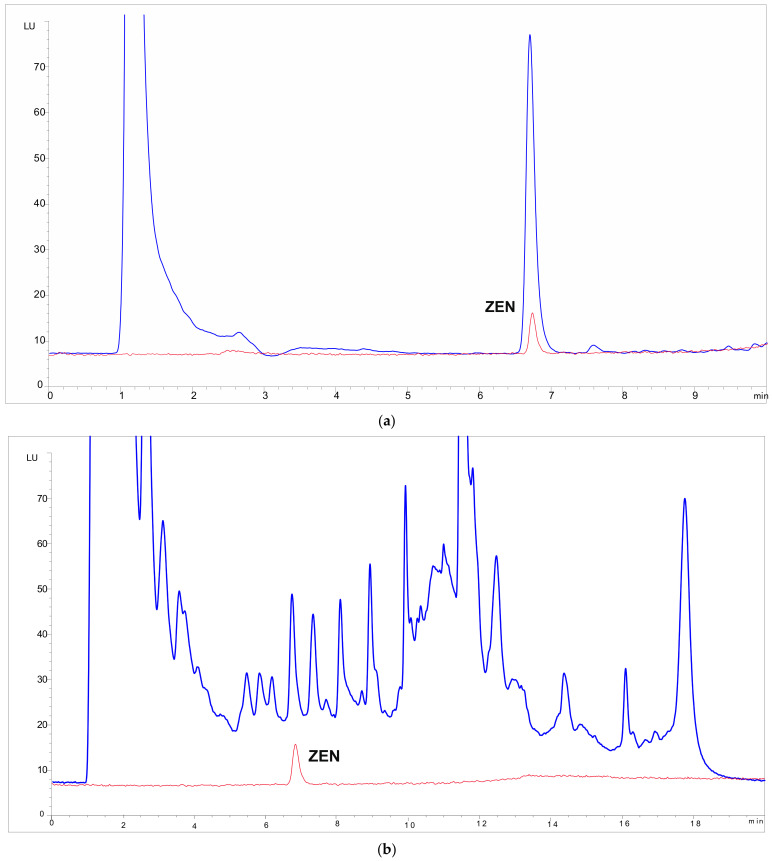
Blue lines: HPLC-FLD chromatograms of the native wheat oil sample-No. 5 from interlaboratory comparison study containing 494 µg/kg ZEN; red lines: ZEN standard solution in acetonitrile (60 ng/mL). Sample preparation according to (**a**) RCHC-SPE method with hydrazine-functionalized material SC-1, and (**b**) official standard method EN 16924:2017 (HPLC-FLD part).

Due to the efficient purification step of the RCHC-SPE method, there is a significant advantage over the standard method EN 16924, which is based on simple LLE using an alkaline mixture for extraction (methanol/10 g/L aqueous ammonium hydrogen carbonate, 9:1 *v*/*v*) without any clean-up. Especially for native oils, the increased matrix load can lead to strong interferences in the FLD chromatograms that do not allow the quantification of ZEN in these samples. Figure 4 shows the FLD chromatograms of the native wheat oil sample-No. 5 from ILC using the new RCHC-SPE method and the current standard method EN 16924.

While the FLD-chromatogram of the RCHC-SPE method (Figure 4a) is quantifiable without any interferences, the chromatogram resulting from sample preparation according to EN 16924 (Figure 4b) shows broad interfering fluorescence signals. This is not only a problem for ZEN quantification, but also for overloading the HPLC-FLD system, resulting in shortened lifetime of analytical equipment, especially the HPLC columns, and increased baselines. The reason for the significantly different chromatograms in RCHC-SPE and EN 16924 sample preparation is obvious. The high methanol content of 90% in the extraction solvent used for EN 16924 leads to the extraction of a wide range of matrix compounds from the oil sample. Due to the lack of a clean-up step, these (fluorescent) matrix compounds are transferred directly to the HPLC-FLD system, where they cause interference. In the RCHC-SPE method, the ZEN analyte covalently bound to the hydrazine solid phase can be efficiently purified. For this purpose, three washing steps with solvents of increasing polarity (n-heptane, ethyl acetate, and methanol) are used, which allows efficient removal of interfering matrix compounds. After washing, ZEN can be decoupled from solid phase, analyzed by HPLC-FLD without matrix interference and thus reliably quantified. This advantage of RCHC-SPE over EN 16924 is particularly important for the analysis of matrix-rich native oils.

Before starting the study, several target criteria were defined that the new method should fulfill. Table 4 summarizes these parameters together with the obtained results of the RCHC-SPE method. All criteria could be achieved considering the results of the ILC. A re-activation of the RCHC-SPE is possible for at least five times, however, the number of cycles depends on the matrix load of the applied oil samples.

## 3. Conclusions

In summary, a reliable and selective method based on reversible covalent hydrazine chemistry (RCHC) was developed for the analysis of zearalenone (ZEN) in edible vegetable oils. The solid phase, consisting of hydrazine-functionalized silica, enables a highly efficient extraction and clean-up procedure, which is a prerequisite for controlling the established maximum levels for ZEN in refined maize oils, but also to analyze matrix-rich native oils. For native edible oils in particular, the RCHC-SPE method was shown to be superior to the official standard method EN 16924 (HPLC-FLD part), which lacks selectivity. Important method performance parameters such as ZEN recovery, repeatability, and reproducibility were determined in an interlaboratory comparison study (ILC), with six edible vegetable oils meeting the criteria of Commission Regulation (EC) No 401/2006. Due to the high efficiency of the RCHC-SPE, only few matrix components enter the HPLC-FLD system, which extends the lifetime of the analytical equipment. The re-useability of the RCHC-SPE cartridges contributes to an eco-friendly method and reduced analysis costs. In a future phase, further savings in handling, time, and costs can be achieved by automation of the process. If widely accepted, the RCHC-SPE method also has the long-term potential to replace the current standard method EN 16924 for routine analysis of ZEN in edible vegetable oils.

## 4. Materials and Methods

### 4.1. Chemical Reagents

A certified Biopure^®^ standard of ZEN (100 µg/mL) in acetonitrile supplied by Romer Labs^®^ (Tulln, Austria) was used to prepare calibration solutions for the quantification of ZEN in edible oils and was provided to the ILC participants for calibration. A certified Biopure^®^ solution of U-[^13^C_18_]-ZEN (25 µg/mL) obtained from Romer Labs^®^ was used as internal standard (ISTD) for the HPLC-MS/MS reference method. Deionized water was supplied by a Seralpur PRO 90CN (Ransbach-Baumbach, Germany). All standard chemicals were of p. A. grade and all solvents HPLC grade.

### 4.2. Hydrazine-Functionalized Materials

Three silica and six polymer-based hydrazine-functionalized materials were included in this study. The tosyl hydrazine silica materials SiliaBond^®^ (SC-1, SC-3) were supplied by SiliCycle^®^ Inc. (Quebec City, QC, Canada), and SC-3 was purchased from Biotage (Uppsala, Sweden). Different polystyrene-divinylbenzene (PS/DVB)-based hydrazine materials were used: the polymer-bound sulfonyl hydrazine (PS-1) consisting of macroporous PS/DVB was obtained from Sigma-Aldrich (Steinheim, Germany), the polymer-bound tosyl hydrazine PS-TsNHNH2 (PS-2) was supplied by Biotage (Uppsala, Sweden), and PS-3 was obtained from Alfa Aesar (Massachusetts, USA). Three further hydrazine-functionalized PS/DVB (PS-4 to PS-6) were custom materials synthesized by Applichrom (Oranienburg, Germany) according to the protocol of Emerson and colleagues [17]. While small PS/DVB particles (5–20 µm) were required for SPE-HPLC online coupling, larger particles (20–80 µm) were preferred for manual SPE to reduce backpressure. Since a pore diameter of 100 Å was initially found to be suitable (PS-5), two more materials (PS-4 and PS-6) with smaller/larger pore diameters were synthesized to cover a wider range.

### 4.3. Edible Vegetable Oil Samples

For the validation of the developed RCHC-SPE method, six commercial edible oil samples were purchased in Germany, five maize germ oils, and one wheat germ oil. Four of the maize oils were refined oils, and one of them the certified reference material ERM^®^-BC715 was produced at BAM. In addition, two native oils (No. 4, No. 5 in Table 3) were included. While the maize oils were analyzed at their natural ZEN levels, the native wheat oil was spiked to a ZEN target value of 500 µg/kg. Spiking was performed by adding 320 µg of ZEN (dissolved in acetonitrile) to a flask. After removal of the solvent (acetonitrile) 640 g of wheat oil was added gravimetrically to obtain 500 µg/kg. The flask was shaken and ultrasonicated for 30 min to re-dissolve ZEN completely. All oil samples were stored tightly closed at 4 °C in a dark place. For the ILC, 60 bottles of 10 mL were prepared of each oil sample. Six out of the sixty bottles were used for homogeneity study and reference value determination using SIDA-HPLC-MS/MS. The homogeneity data were evaluated analysis of variance (ANOVA). As expected, no significant inhomogeneities were observed.

### 4.4. RCHC-SPE Method with HPLC-FLD

*Sample preparation:* The optimized RCHC-SPE sample preparation is described above as significant part of the method development.

*HPLC-FLD measurement:* HPLC-FLD analyses were performed using an Agilent 1200 HPLC tower equipped with Agilent 1200 DAD and FLD detectors (Agilent, Böblingen, Germany). A Gemini^®^ NX C18 110 Å analytical column (150 mm × 2 mm, 3 µm particle size; Phenomenex, Torrance, CA, USA) was used in combination with the respective guard column (4 mm × 2 mm). The following HPLC parameters were employed: oven temperature: 40 °C, injection volume: 20 µL, flow rate: 0.3 mL/min, solvent A: water + 0.1% (*v*) formic acid, solvent B: acetonitrile + 0.1% (*v*) formic acid. A linear gradient was applied from 45% to 60% B between 0.0 and 6.0 min. The system was run with 100% B between 6.1 and 13.0 min and re-equilibrated for 7 min (total run time: 20 min). FL detection was operated with λ_Ex_ = 274 nm and λ_Em_ = 456 nm. Data acquisition was performed using Chemstation software (Agilent).

*Calibration and Quantification*: A stock solution of ZEN in acetonitrile was gravimetrically prepared from the certified standard and stored at −20 °C. Calibrations solutions were prepared by weighing variable amounts of ZEN stock solution, removing acetonitrile in a gentle stream of nitrogen and gravimetric addition of water:acetonitrile:formic acid 50:50:0.1 (*v*/*v*/*v*). Calibrations and samples were analyzed together in one measurement sequence. The ZEN content of edible oil samples was quantified applying a six-point calibration curve after linear regression established in a range of 10−550 μg/kg by analyzing each calibration and sample solution in duplicate.

### 4.5. SIDA-HPLC-MS/MS (Reference Method)

A volume of 200 μL ISTD of U-[^13^C_18_]-ZEN stock solution (acetonitrile) was weighed into a 15 mL reaction tube. After evaporation to dryness at 50 °C, 0.5 mL of the oil sample was added gravimetrically and diluted with 0.5 mL n-hexane. ZEN was extracted from the sample with 5 mL methanol:water 9:1 (*v*/*v*) for 30 min by horizontal shaking (400 min^−1^). The tubes were centrifuged at 2400 rpm (1378× *g*) for 10 min. A volume of 1 mL of the upper (methanolic/water) layer was transferred into an HPLC vial. The solution was evaporated to dryness and re-dissolved in 0.4 mL of water:acetonitrile:formic acid 62:38:0.1 (*v*/*v*/*v*).

*HPLC-MS*/*MS Measurement*: HPLC-MS/MS analyses were performed on an Agilent 1200 series HPLC hyphenated to an API 4000 QTRAP^®^ hybrid mass spectrometer (Sciex, Foster City, CA, USA). The same HPLC column was used as described above for the RCHC-SPE method. Chromatographic conditions were as follows: oven temperature: 50 °C, injection volume: 10 µL, flow rate: 0.3 mL/min, solvent A: water + 0.1 % (*v*) formic acid, solvent B: acetonitrile + 0.1 % (*v*) formic acid. The following gradient was chosen: 38% B between 0.0 and 15.0 min, 95% B between 15.1 and 19.0 min, 38% A between 19.1 and 27.0 min (re-equilibration). The mass spectrometer was operated in MRM mode with ESI (negative) detection, monitoring mass transitions for native ZEN (*m*/*z*) 317.1 → 131.1 (quantifier) and 317.1 → 175.0 (qualifier). For the U-[^13^C_18_]-ZEN (*m*/*z*), 335.2 → 140.2 was recorded. Ion source parameters were as follows: ion spray voltage: −4000 V, desolvation temperature: 500 °C, ion source gas: 1:50 arbitrary units (a.u.), ion source gas: 2: 50 a.u., curtain gas: 20 a.u. The optimized compound specific MRM parameters were: declustering potential: −80 V entrance potential: −10 V, dwell time: 50 ms. Collision energy and collision cell exit potential were differing for quantifier/qualifier/U-[^13^C_18_]-ZEN and were −42/−40/−42 V and −13/−13/−7 V, respectively. Data acquisition was performed using Analyst 1.5.2 software (AB Sciex, Foster City, CA, USA).

*Calibration and Quantitation:* While the general scheme described for the RCHC-SPE method was also applied for SIDA-HPLC-MS/MS, the ISTD was present in all measurement solutions.

## Figures and Tables

**Table 1:** Hydrazine-functionalized materials (SC: silica; PS/DVB: polystyrene-divinylbenzene).

Code	Solid Phase	Particle Diameter (µm)	Pore Diameter (Å)	Coupling Rate Constant (×10^−4^ min^−1^)
**SC-1**	Silica	40–63	55–65	865 ± 27
**SC-2**	Silica	60	40	≈0
**SC-3**	Silica	5	59	790 ± 17
**PS-1**	PS/DVB	200–595	614	730 ± 17
**PS-2**	PS/DVB	75–150	- ^a^	429 ± 10
**PS-3**	PS/DVB	37–74	- ^a^	≈0
**PS-4**	PS/DVB	20–80	60	750 ± 35
**PS-5**	PS/DVB	20–80	100	812 ± 25
**PS-6**	PS/DVB	20–80	500	451 ± 27

^a^ no information available from supplier.

**Table 2:** Coupling and decoupling properties of 5 HFM candidates for RCHC-SPE material. Values of the coupling experiment represent the relative amount of ZEN in solution not coupled to HFM; values of the decoupling study represent ZEN (in %) in solution released from HFM; values are means of three replicates.

Coupling					
Time (min)	ZEN in Solution (%)				
	SC-1	SC-3	PS-1	PS-4	PS-5
0	100	100	100	100	100
30	5	10	19	15	8
60	0	5	7	9	0
90	0	0	2	4	0
120	0	0	0	2	0
**Decoupling**					
0	0	0	0	0	0
30	95	79	80	84	70
60	94	83	78	88	78
90	93	88	82	89	85
120	95	95	80	93	88

**Table 3:** Results of the ILC for method validation to determine ZEN in edible vegetable oils.

Edible Vegetable Oil Sample		No. of Labs	ILC Mean (µg/kg)	Reference Value (µg/kg)	ZEN Recovery (%)	RSD_r_ ^b^ (%)	RSD_R_ ^c^ (%)
#1	Refined maize oil	10	39	47	83	7.0	17
#2	Refined maize oil	10	157	188	83	6.2	18
#3	Refined maize oil (ERM-BC715)	10	292	362	81	7.5	22
#4	Native maize oil	10	84	98	85	9.7	17
#5	Native wheat oil	10	399	494	81	4.8	17
#6	Refined maize oil (“Blank”)	10	(4.9) ^a^	3.3	-	-	-

^a^ Indicative value; ^b^ Repeatability; ^c^ Reproducibility.

**Table 4:** Technical target criteria and achieved results for development of an RCHC-SPE method.

Parameter	Targeted	Achieved
Material of RCHC-SPE cartridge	PP/PE	PP/PE
Volume of RCHC-SPE cartridge	1, 3, or 6 mL	1 mL
Amount of HFM per cartridge	100–500 mg	400 ± 2 mg SC-1
ZEN capacity of RCHC-SPE cartridge	≥0.2 µg	0.5 µg
Application range ^a^	50–500 µg/kg	47–494 µg/kg
ZEN recovery ^b^	70–120%	83%
Repeatability (RSD_r_) ^b^	≤25	7.0
Reproducibility (RSD_R_) ^b^	≤40	18
Regeneration cycles of RCHC-SPE	>5 cycles	>5 cycles

^a^ Application range of 50–500 µg/kg is based on standard procedure [10] and the European maximum level for ZEN in edible vegetable oils of 400 µg/kg. ^b^ Target value according to the requirements of European Regulation (EC) No 401/2006 [16].

## Data Availability

Not applicable.
